# Spermine-mediated metabolic homeostasis improves growth and stress tolerance in creeping bentgrass (*Agrostis stolonifera*) under water or high-temperature stress

**DOI:** 10.3389/fpls.2022.944358

**Published:** 2022-08-11

**Authors:** Zhou Li, Bizhen Cheng, Xing Wu, Yan Zhang, Guangyan Feng, Yan Peng

**Affiliations:** Department of Turf Science and Engineering, Sichuan Agricultural University, Chengdu, China

**Keywords:** abiotic stress, metabolites, metabolic pathways, osmotic adjustment (OA), polyamine (PA), signal transduction

## Abstract

Plants have developed diverse defense strategies to reduce the detrimental effects of a wide range of environmental stresses. The objectives of this study were to explore the function of spermine (Spm) on mediating growth and physiological changes in water homeostasis, photosynthetic performance, and oxidative damage and to further examine the regulatory mechanism of Spm on global metabolites reprogramming and associated metabolic pathways in horticultural creeping bentgrass (*Agrostis stolonifera*) under water and heat stresses. The 21-days-old plants were pretreated with or without 100 μM Spm for 3 days and then subjected to water stress (17% polyethylene glycol 6000), high-temperature stress (40/35°C, day/night), or normal condition (control without water stress and heat stress) for 18 days. Results demonstrated that exogenous application of Spm could significantly increase endogenous polyamine (PAs), putrescine (Put), spermidine (Spd), and Spm contents, followed by effective alleviation of growth retardant, water imbalance, photoinhibition, and oxidative damage induced by water and heat stress. Metabolites' profiling showed that a total of 61 metabolites were differentially or commonly regulated by Spm in leaves. Spm upregulated the accumulation of mannose, maltose, galactose, and urea in relation to enhanced osmotic adjustment (OA), antioxidant capacity, and nitrogen metabolism for growth maintenance under water and heat stress. Under water stress, Spm mainly induced the accumulation of sugars (glucose-1-phosphate, sucrose-6-phosphate, fructose, kestose, maltotriose, and xylose), amino acids (glutamic acid, methionine, serine, and threonine), and organic acids (pyruvic acid, aconitic acid, and ketoglutaric acid) involved in the respiratory pathway and myo-inositol associated with energy production, the ROS-scavenging system, and signal transduction. In response to heat stress, the accumulation of alanine, glycine, gallic acid, malic acid, or nicotinic acid was specifically enhanced by Spm contributing to improvements in antioxidant potency and metabolic homeostasis. This study provides novel evidence of Spm-induced,tolerance to water and heat stresses associated with global metabolites reprogramming in favor of growth maintenance and physiological responses in horticultural plants.

## Introduction

Abiotic stresses such as drought and high-temperature stress cause huge economic losses in horticulture. Predictably, the frequency and severity of drought and heat stress will further increase in the future due to global climate change (Cayan et al., 2010; Sun et al., 2019). Plants adapt to stressful environments by regulating physiological, biochemical, and phenotypic responses at molecular, enzymatic, and metabolic levels (Gong et al., 2020; Jing et al., 2021; Li et al., 2021). Although the analysis and identification of stress-responsive genes and proteins are still the mainstream research in plant species, the role of metabolites in stress tolerance is coming into notice in recent years (Ghatak et al., 2018). Increasing studies proved that global metabolites accumulation and transformation not only affected crop product and quality but also affected stress tolerance. Some of them are beneficial for enhanced stress tolerance, such as most of the amino acids and soluble sugars, but others like quinones and aldehydes overaccumulation are toxic to plants under stressful environments (Srivastava et al., 2017; Martínez-Noël and Tognetti, 2018; Batista-Silva et al., 2019). It has been demonstrated that the global reprogramming of metabolites was involved in adaptive responses to drought stress in different *Lotus genus* species by using comparative metabolomics (Sanchez et al., 2012). Non-protein amino acids, such as γ-aminobutyric acid (GABA), could mitigate heat and drought damage by altering primary and secondary metabolites in creeping bentgrass (*Agrostis stolonifera*) based on the analysis of non-targeted metabolomics (Li et al., 2016a, 2017a). Therefore, the study of the relations between changes in metabolites and stress tolerance is essential for a better understanding of the stress-adaptive mechanisms in different plant species.

Polyamines (PAs) including putrescine (Put), spermidine (Spd), and spermine (Spm) are known as low-molecular-weight plant growth regulators (PGRs) exhibiting strong biological activity in plants. Spm is a superlative form of PA which is synthesized from a lower form of PA, such as Put and Spd, and Spm can also be degraded into Spd and Put in plants (Chen et al., 2019). It has been widely reported about the function of PAs in regulating plant growth, development, senescence, and stress tolerance in various plant species (Chen et al., 2019). Put and Spd pretreatment mitigated cadmium damage to wheat (*Triticum aestivum*) by maintaining hormonal balance and antioxidant homeostasis (Tajti et al., 2018). Exogenously applied Spd enhanced endogenous PAs accumulation and tolerance to water stress in white clover (*Trifolium repens*) in relation to the improvement in antioxidant and proline metabolisms (Li et al., 2016b), whereas the inhibition of endogenous PAs biosynthesis could significantly decrease drought tolerance of white clover (Li et al., 2018). PAs could alleviate high-temperature stress by modulating starch and PAs accumulation and metabolism in rice (*Oryza sativa*) (Fu et al., 2019). Heat tolerance was established with the application of exogenous Spd in rice closely related to the enhancement of antioxidative and glyoxalase systems (Mostofa et al., 2014). However, the contribution of Spm to drought and heat tolerance associated with changes in global metabolites and relevant metabolic pathways is poorly documented in plants.

Mesophytes including most of the horticultural plants, such as creeping bentgrass, are susceptible to stressful environments, leading to a decrease in quality and economic losses (Cramer et al., 2011). In this study, we focused on effects of exogenous application of Spm on changes in endogenous PAs content, physiological responses, including photosynthesis, osmotic adjustment (OA), and oxidative damage, and extensive organic metabolites based on metabolomics in leaves of creeping bentgrass under normal, drought, and heat conditions. Current findings will help to better understand critical roles of PAs and metabolites in cold-season creeping bentgrass in response to drought and high-temperature environments.

## Materials and methods

### Plant material and treatments

Seeds (cv. Penncross at a rate of 6 g·m^−2^) were germinated in rectangular plastic containers (25 cm in length, 15 cm in width, and 10 cm in height) filled with quartz sand in growth chambers (21/18°C (day/night), 65% relative humidity, and 700 μmol·m^−2^·s^−1^ PAR). After germinating in distilled water for 8 days, seedlings were irrigated with Hoagland's solution (Hoagland and Arnon, 1950) for 14 days. For hydroponic cultivation, seedlings were removed from quartz sand and suspended through small holes in Styrofoam boards that could float on the nutrient solution. After being transplanted in Hoagland's solution for 7 days for the acclimation to the hydroponic cultivation, seedlings were grown in the Hoagland's solution without (as control) or with 100 μmol/L Spm (as +Spm, purity ≥ 99.0%, Art. No. 85590, Sigma-Aldrich) for 3 days and then all plants were moved to a new Hoagland's solution without Spm. Three conditions were set up: (1) normal condition: Spm-pretreated and untreated plants grew in a normal Hoagland's solution and were placed in a normal growth chamber (mentioned above) for 18 days; (2) water stress: Spm-pretreated and untreated plants grew in a Hoagland's solution containing 17% polyethylene glycol 6000 (PEG-induced water stress) and were placed in a normal growth chamber for 18 days; (3) heat stress: Spm-pretreated and untreated plants grew in the normal Hoagland's solution and were placed in a high-temperature growth chamber [40/35°C (day/night), 65% relative humidity, and 700 μmol·m^−2^·s^−1^ PAR] for 18 days. All solutions were refreshed every day. Spm-pretreated and untreated plants were completely arranged in growth chambers. Four independent biologic replicates (four containers) for each treatment including 20 plants were used for the analysis of physiological parameters or metabolome.

### Measurements of physiological parameters and endogenous polyamines content

The determination of chlorophyll (Chl) content, relative water content (RWC), hydrogen peroxide (H_2_O_2_), malondialdehyde (MDA) content, electrolyte leakage (EL), endogenous PAs, and relative growth rate (RGR) were performed based on the method of Amnon (1949); Barrs and Weatherley (1962); Blum and Ebercon (1981); Dhindsa et al. (1981); Velikova et al. (2000); Duan et al. (2008), and Ma et al. (2012), respectively. Specific measurement procedures have been clearly described in our previous study (Li et al., 2016b). For the determination of OA, fresh leaves were hydrated in deionized water for 12 h and fully turgid leaves were frozen in liquid nitrogen for 10 min. When frozen leaves were thawed in an ice bath, the sap in the leaves was pressed and then inserted (10 ml) into an osmometer (Wescor, Inc., Logan, UT) to get osmolality (mmol kg^−1^). The osmotic potential (OP) was calculated based on the formula OP = –([osmolality] × [0.001] × [2.58]). The OA was calculated based on the formula OA = OP (control leaves under the normal condition) – OP (stressed leaves) (Blum, 1989). A portable photosynthesis system (CIRAS-3, PP Systems, USA) was used for detecting net photosynthesis rate (Pn) and water use efficiency (WUE). The leaf chamber (400 μl L^−1^ CO_2_ and 800 μmol photon m^−2^ red and blue light) was overspread with a single layer of leaves and readings were recorded after the readings were stable. The protein carbonyl content was detected using the assay kit that was purchased from the Suzhou Comin Biotechnology Co., Ltd., Suzhou, China, according to the manufacturer's instructions.

### Metabolite extraction and quantification

The metabolome was detected using GC-TOFMS. The metabolite extraction, separation, and quantification were conducted according to the methods of Roessner et al. (2000) and Qiu et al. (2009) with some modifications that were clearly described in our previous study (Li et al., 2020a).

### Statistical analysis

The analysis of variance (SAS 9.1, SAS Institute, Cary, NC, USA) was used to determine the significance of all measured parameters. Differences between treatment means were tested using Fisher's protected least significance (LSD) test at a 0.05 probability level.

## Results

### Effects of spermine priming on changes in endogenous polyamines

**Figure 1:**
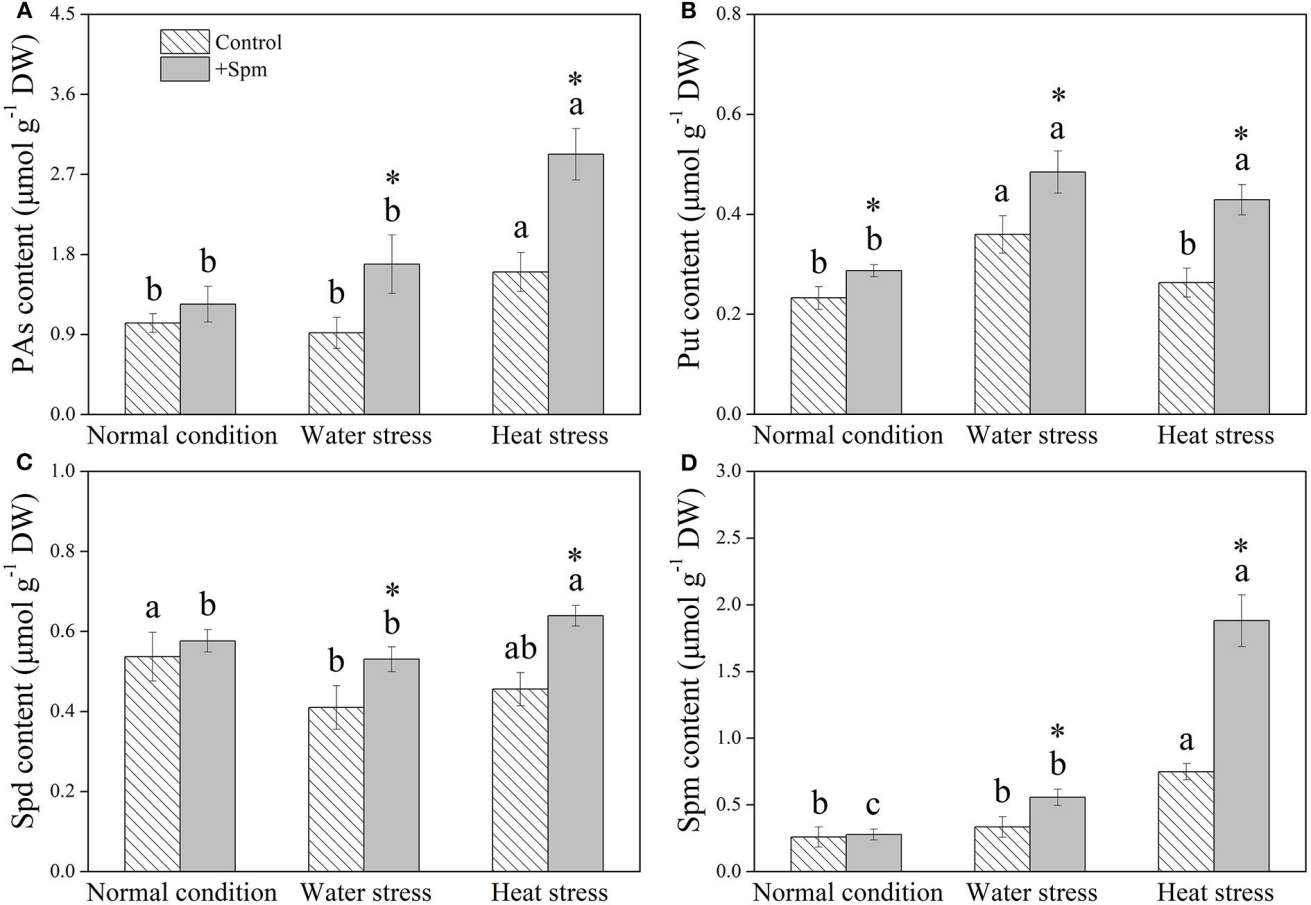
Changes in endogenous **(A)** polyamines (PAs), **(B)** putrescine (Put), **(C)** spermidine (Spd), and **(D)** spermine (Spm) in response to Spm application under different conditions. Vertical bars indicate ±SE of mean (*n* = 4). Different letters above striated or gray columns indicate significant differences for a particular treatment (control or +Spm) for comparison across the normal condition, water stress, and heat stress; the asterisk “*” indicates significant differences between the control and the +Spm treatments under one particular condition (normal condition, water stress, or heat stress) based on LSD (*P* < 0.05). Total PA content was calculated by the sum of Put, Spd, and Spm contents.

Spm priming did not affect PA content in leaves under normal condition, but Spm-pretreated plants exhibited an 84 or 82% increase in PA content than non-treated control under water stress or heat stress, respectively (Figure 1A). The application of exogenous Spm significantly increased Put content under normal condition, water stress, and heat stress (Figure 1B). A 29 or 40% increase in Spd content was observed in +Spm treatment as compared to that in control under water stress or heat stress, respectively (Figure 1C). Spm-treated plants exhibited significantly higher endogenous Spm content than untreated plants under water stress and heat stress (Figure 1D). For untreated plants, Spm significantly accumulated under heat stress, but did not significantly increase under water stress as compared to the control. For Spm-treated plants, Spm significantly accumulated under water stress and heat stress (Figure 1D).

### Effects of spermine priming on changes in growth, water status, and photosynthesis

**Figure 2:**
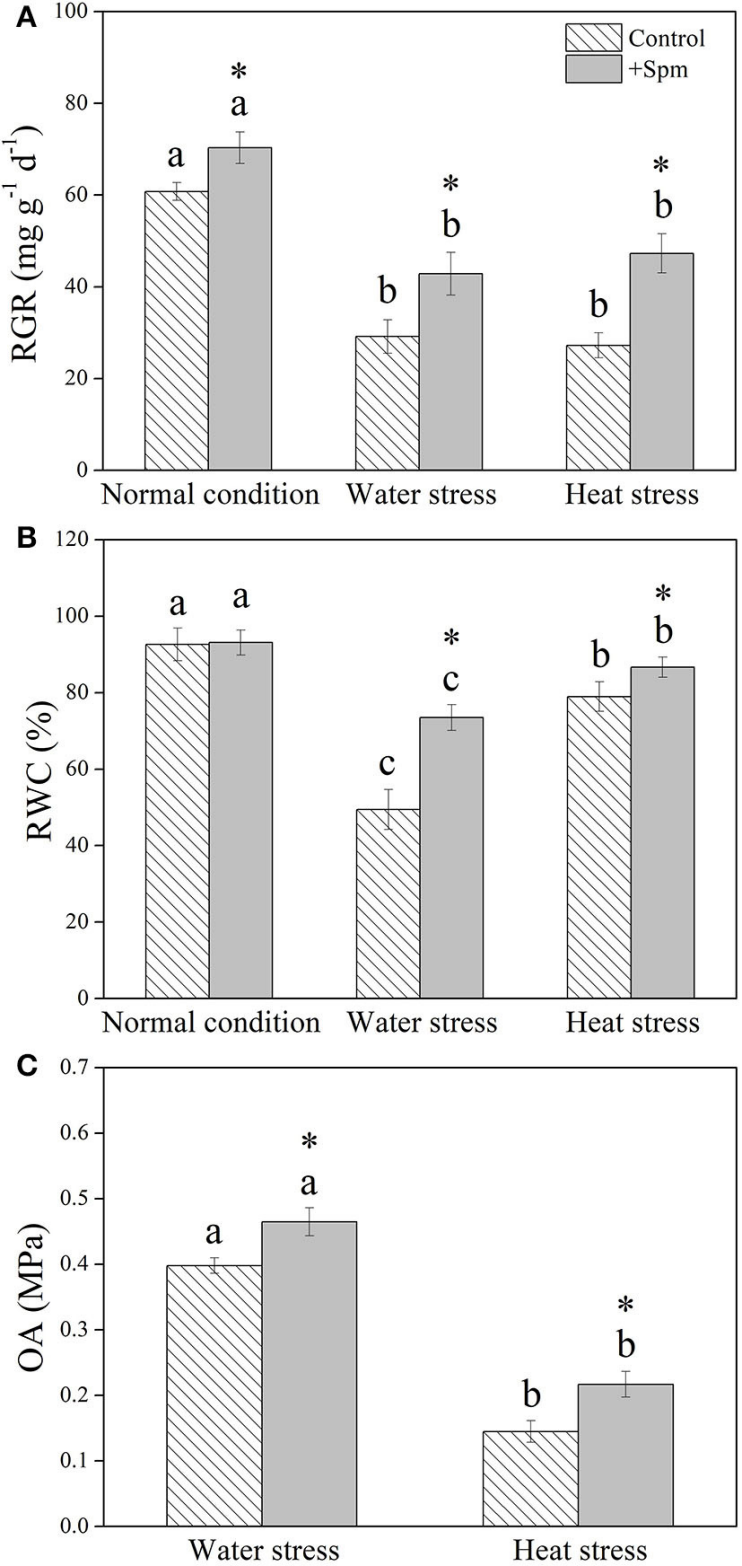
Changes in **(A)** relative growth rate (RGR), **(B)** relative water content (RWC), and **(C)** osmotic adjustment (OA) in response to Spm application under different conditions. Vertical bars indicate ±SE of mean (*n* = 4). Different letters above striated or gray columns indicate significant differences for a particular treatment (Control or +Spm) for comparison across the normal condition, water stress, and heat stress; the asterisk “*” indicates significant differences between the control and the +Spm treatments under one particular condition (normal condition, water stress, or heat stress) based on LSD (*P* < 0.05).

**Figure 3:**
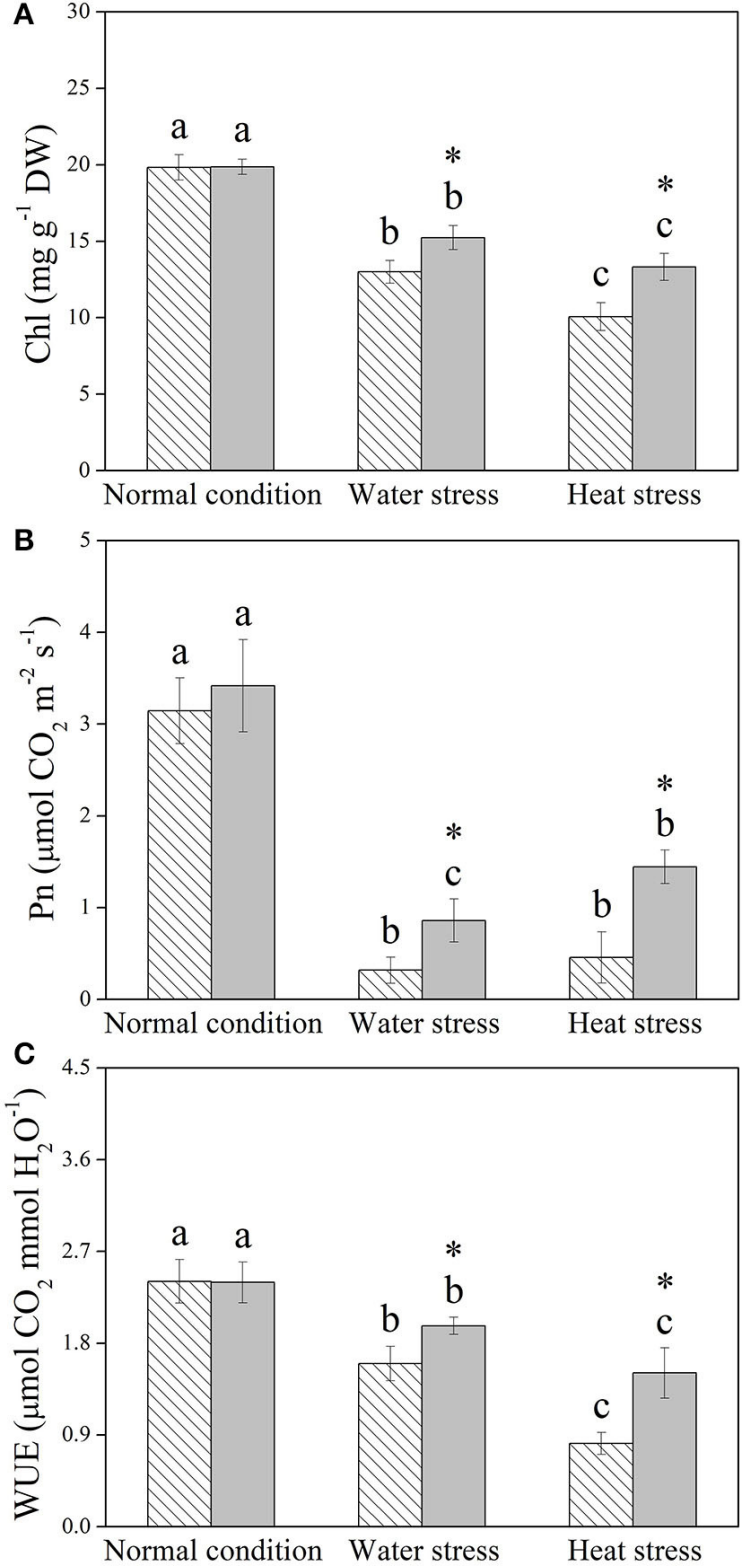
Changes in **(A)** chlorophyll (Chl) content, **(B)** net photosynthetic rate (Pn), and **(C)** water use efficiency (WUE) in response to Spm application under different conditions. Vertical bars indicate ±SE of mean (*n* = 4). Different letters above striated or gray columns indicate significant differences for a particular treatment (Control or +Spm) for comparison across the normal condition, water stress, and heat stress; the asterisk “*” indicates significant differences between the control and the +Spm treatments under one particular condition (normal condition, water stress, or heat stress) based on LSD (*P* < 0.05).

Water stress and heat stress significantly decreased the RGR of the control and +Spm treatments, but the +Spm treatment showed significantly higher RGR than the control under normal condition, water stress, and heat stress (Figure 2A). The application of exogenous Spm significantly alleviated the decline in leaf RWC induced by water stress and heat stress (Figure 2B). As compared to untreated control, Spm-pretreated plants had a 17 or 50% increase in OA under water stress or heat stress, respectively (Figure 2C). Chl content, Pn, and WUE also significantly declined in response to water stress and heat stress in both of Spm-pretreated and non-treated plants, whereas Spm-pretreated plants showed significantly higher Chl content, Pn, and WUE than non-treated plants under water stress and heat stress (Figures 3A–C).

### Effects of spermine priming on changes in oxidative damage and cell membrane stability

**Figure 4:**
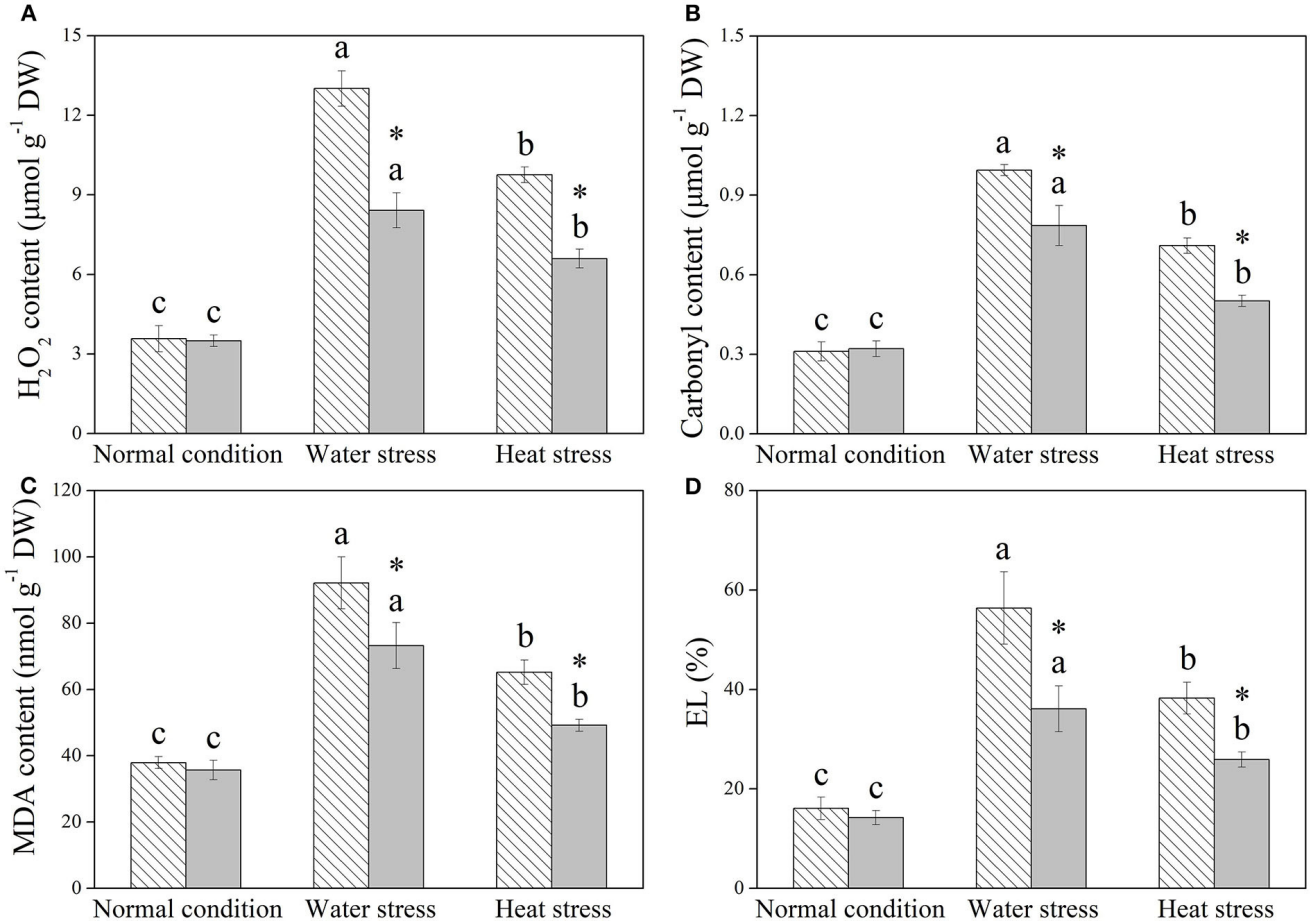
Changes in endogenous **(A)** hydrogen peroxide (H_2_O_2_), **(B)** carbonyl content, **(C)** malonaldehyde (MDA), and **(D)** electrolyte leakage (EL) in response to Spm application under different conditions. Vertical bars indicate ±SE of the mean (*n* = 4). Different letters above striated or gray columns indicate significant differences for a particular treatment (Control or +Spm) for comparison across the normal condition, water stress, and heat stress; the asterisk “*” indicates significant differences between the control and the +Spm treatments under one particular condition (normal condition, water stress, or heat stress) based on LSD (*P* < 0.05).

H_2_O_2_, protein carbonyl content, MDA content, and EL were not significantly affected by Spm priming under normal condition (Figures 4A–D). Water stress and heat stress significantly induced increases in these four parameters in both the control and +Spm treatments; however, Spm priming significantly decreased stress-induced increases in H_2_O_2_, carbonyl, MDA content, and EL in leaves (Figures 4A–D).

### Effects of spermine priming on change in metabolites profiling

**Figure 5:**
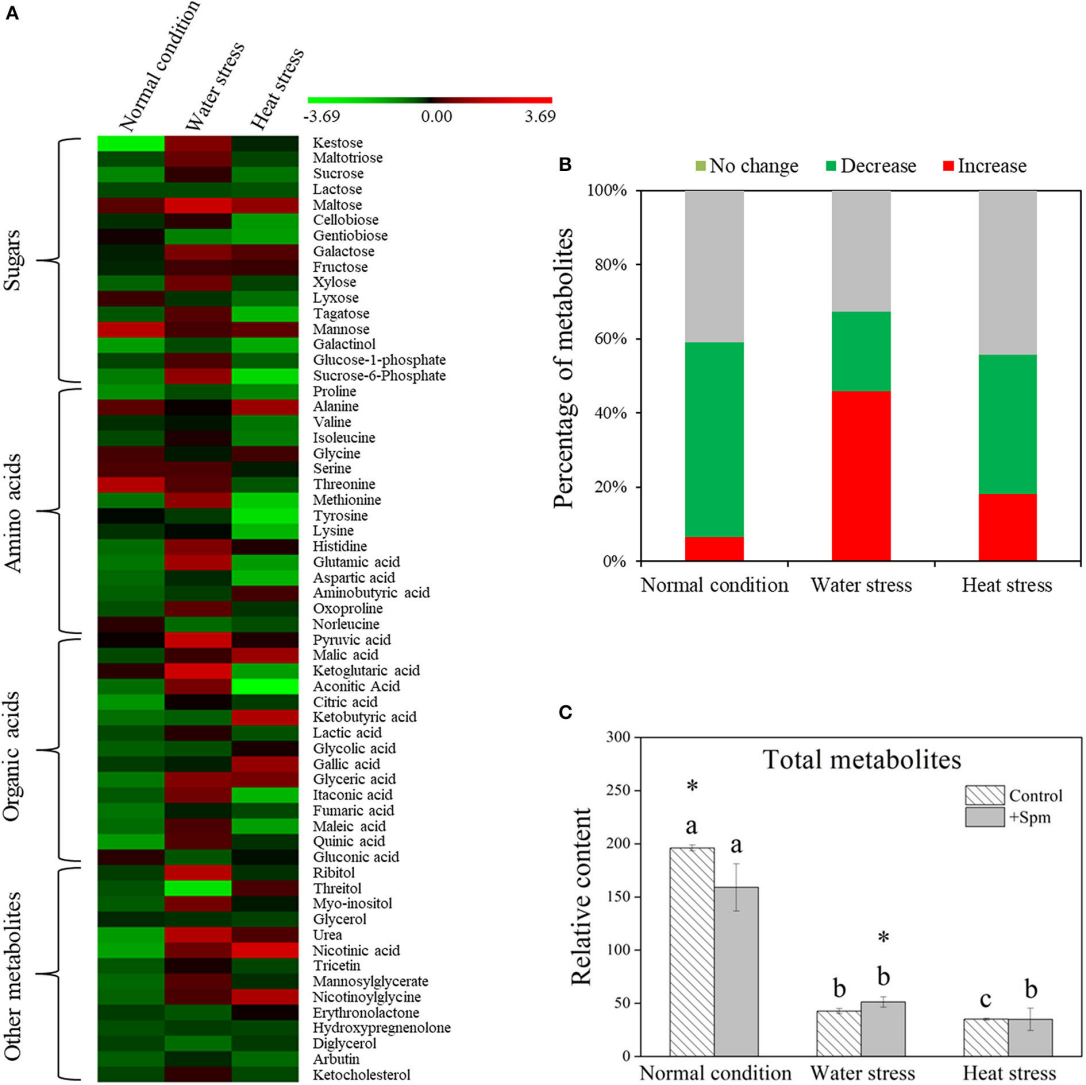
Changes in **(A)** the heatmap of all identified metabolites, **(B)** percentage of upregulated, downregulated, and unchanged metabolites, and **(C)** relative content of total metabolites in response to Spm application under different conditions. Vertical bars indicate ±SE of mean (*n* = 4). Different letters above striated or gray columns indicate significant differences for a particular treatment (Control or +Spm) for comparison across the normal condition, water stress, and heat stress; the asterisk “*” indicates significant differences between the control and the +Spm treatments under one particular condition (normal condition, water stress, or heat stress) based on LSD (*P* < 0.05).

**Figure 6:**
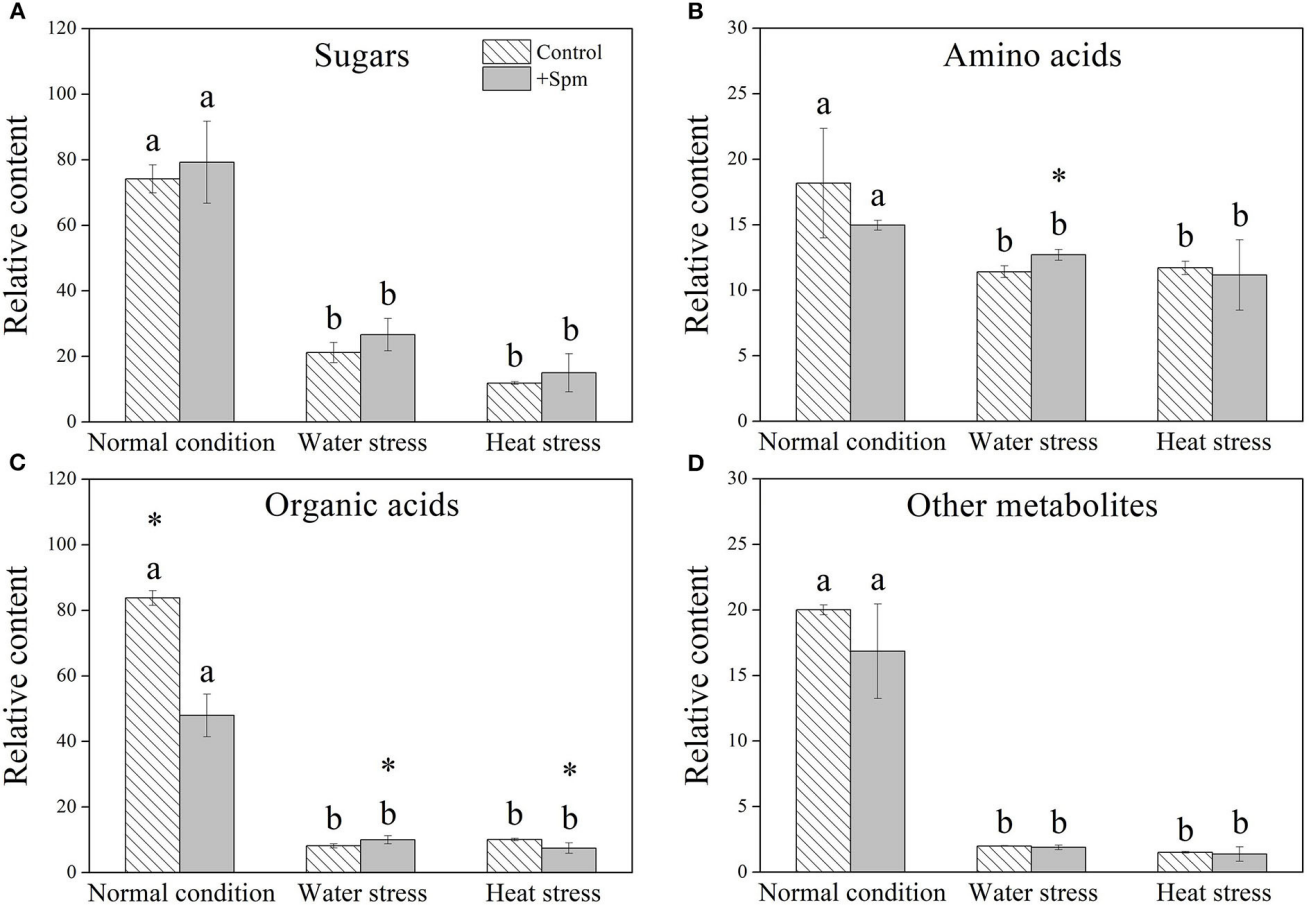
Changes in the relative content of **(A)** sugars, **(B)** amino acids, **(C)** organic acids, and **(D)** other metabolites in response to Spm application under different conditions. Vertical bars indicate ±SE of mean (*n* = 4). Different letters above striated or gray columns indicate significant differences for a particular treatment (Control or +Spm) for comparison across the normal condition, water stress, and heat stress; the asterisk “*” indicates significant differences between the control and the +Spm treatments under one particular condition (normal condition, water stress, or heat stress) based on LSD (*P* < 0.05).

A total of 61 metabolites were identified in leaves of creeping bentgrass under normal condition, water stress, and heat stress (Figure 5A). These metabolites included 16 sugars, 16 amino acids, 15 organic acids, and 14 other metabolites. A heatmap of all metabolites showed downregulation and upregulation as Spm-treated plants compared to untreated plants under normal condition, water stress, or heat stress, respectively (Figure 5A). The ratio of no change, a significant increase, or a significant decline in metabolites was shown in Figure 5B, when Spm-pretreated plants were compared to untreated plants under normal condition, water stress, or heat stress. Water stress or heat stress significantly decreased total metabolites accumulation, and Spm-pretreated plants exhibited significantly higher total metabolites content than untreated plants under water stress (Figure 5C). Similarly, total sugars, amino acids, organic acids, or other metabolites also significantly declined in all plants under water stress and heat stress (Figures 6A–D). The Spm-pretreated plants accumulated significantly more total amino acids and organic acids under water stress (Figures 6B,C).

**Figure 7:**
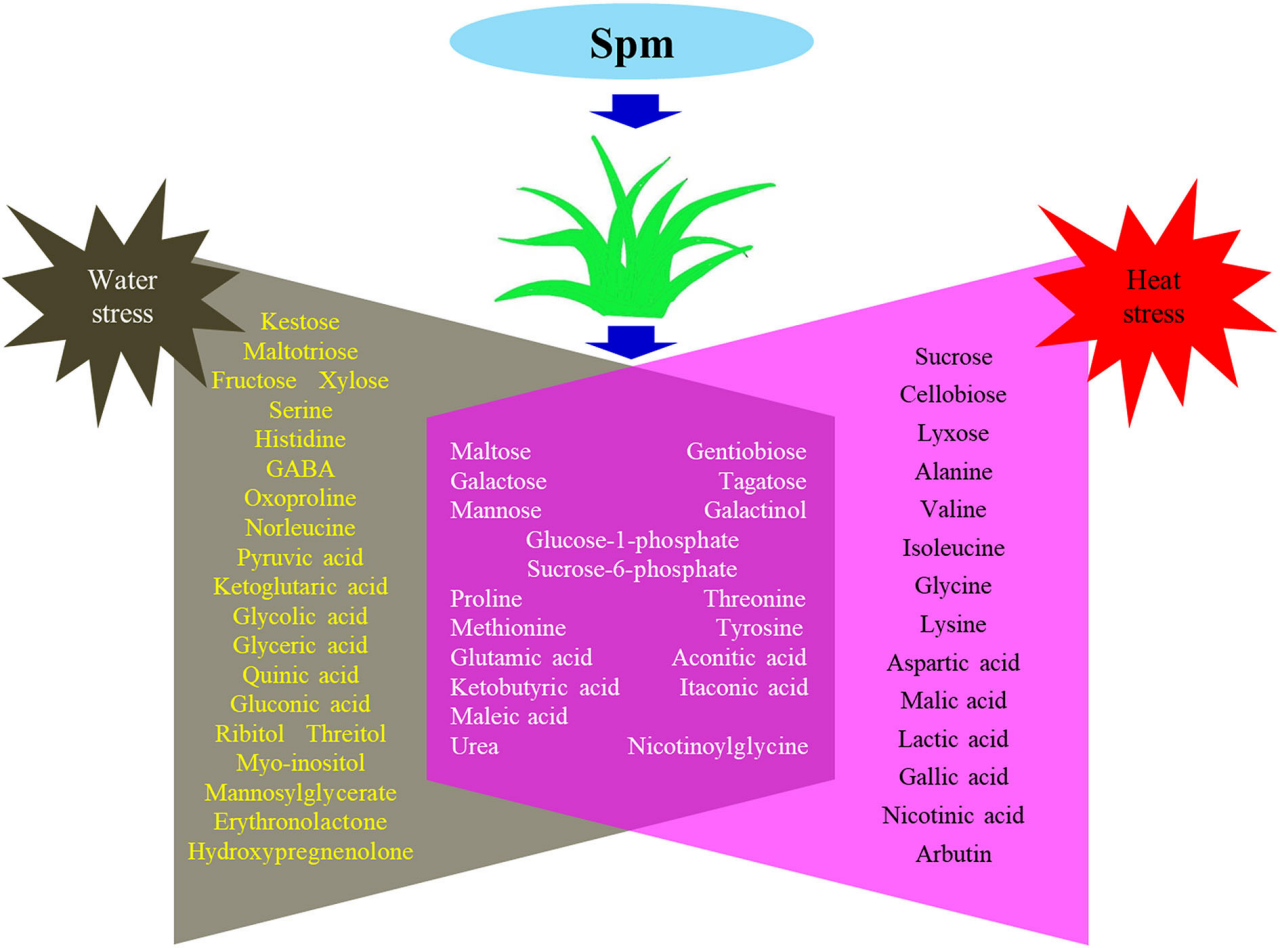
Common and differential metabolites regulated by spermine under water stress and heat stress in leaves of creeping bentgrass.

**Figure 8:**
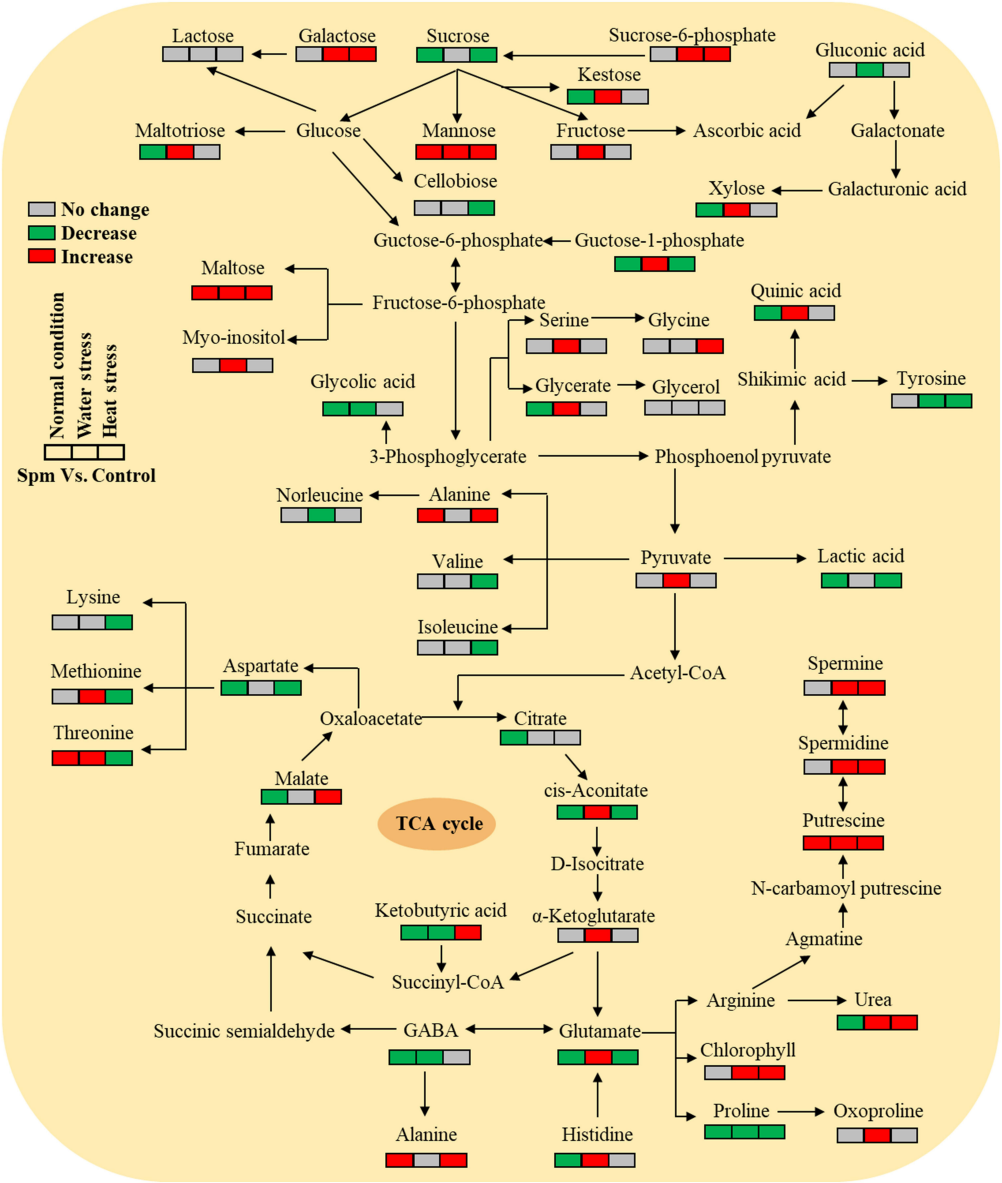
Pathway enrichment analysis of 45 identified metabolites in leaves of creeping bentgrass regulated by spermine under the normal condition, water stress, and heat stress.

Supplementary Figures S1–S4 showed the relative content of 61 metabolites. Among these metabolites, Spm upregulated the accumulation of 10 sugars (kestose, maltotriose, maltose, galactose, fructose, xylose, tagatose, mannose, glucose-1-phosphate, and sucrose-6-phosphate), 5 amino acids (serine, threonine, methionine, histidine, and glutamic acid), 7 organic acids (pyruvic acid, ketoglutaric acid, aconitic acid, glyceric acid, itaconic acid, maleic acid, and quinic acid), and 5 other metabolites (ribitol, myo-inositol, urea, mannosylglycerate, and nicotinoylglycine) under water stress (Supplementary Figures S1–S4). Under heat stress, Spm application improved the accumulation of maltose, galactose, mannose, alanine, glycine, malic acid, ketobutyric acid, gallic acid, urea, nicotinic acid, and nicotinoylglycine (Supplementary Figures S1–S4). As seen in Figure 7, Spm regulated 21 metabolites under water stress. A total of 14 metabolites responded to Spm application under heat stress (Figure 7). Spm induced changes in 19 metabolites in leaves under water and heat stress (Figure 7). Pathway enrichment analysis showed 45 identified metabolites involved in the tricarboxylic acid (TCA) cycle, GABA shunt, and other metabolic pathways (Figure 8).

## Discussion

Increases in endogenous PAs biosynthesis and catabolism were propitious to achieve tolerance to water stress in *Arabidopsis thaliana* (Sen et al., 2018; Sen and Mohapatra, 2022). However, enhanced PA metabolism could also improve the risk of oxidative damage, because PA catabolism was known to generate ROS (Mohapatra et al., 2009). Our current study showed that Spm pretreatment did not significantly affect the accumulation of total PAs, Spd, or Spm and physiological parameters, including RWC, Chl content, Pn, WUE, H_2_O_2_ content, carbonyl content, MDA content, and EL level in leaves of creeping bentgrass under the normal condition, but significantly improved RGR. These findings indicated that an appropriate dose of Spm pretreatment did not cause deleterious effects in creeping bentgrass under the normal condition, and Spm could be catabolized for maintaining growth during 18 days of normal cultivation. In addition, alterations of endogenous PA content under stress conditions are evident in many plant species in relation to drought and heat tolerance. For example, declines in endogenous Put, Spd, and Spm content by the application of a biosynthetic inhibitor of PAs significantly weakened the adaptive response of white clover to water stress (Li et al., 2015b). Significant increases in endogenous Spd and Spm levels induced by exogenous Spd enhanced the heat tolerance of rice (Zhou et al., 2020). Better maintenance of water balance and photosynthesis was associated with Spd-regulated tolerance to water stress in white clover (Li et al., 2015a). The study of Fu et al. (2014) also proved that Spm pretreatment conferred combined heat and drought tolerance in trifoliate orange (*Poncirus trifoliata*) by alleviating oxidative damage. Similar results were found in this study. Spm pretreatment induced significant increases in endogenous Put, Spd, and Spm under water or heat stress, followed by the mitigation of detrimental effects of water stress or heat stress, such as growth inhibition, photoinhibition, water loss, and oxidative damage in creeping bentgrass. Evidently, PA-regulated tolerance to water stress and heat stress were closely related to OA, photosynthetic maintenance, and oxidation-reduction equilibrium in plants. Some common or particular organic metabolites contributing to OA and redox homeostasis in creeping bentgrass in response to water and heat stress are discussed below.

Regulatory effects of Spm on sugar accumulation and metabolism have been reported in many plant species under drought since sugars exhibit the important function of OA, signal transduction, and energy supply (Hasan et al., 2021). For example, exogenous application of Spm improved total soluble sugars accumulation in favor of water relation in soybean (*Glycine max*) (Dawood and Abeed, 2020), white clover (Li et al., 2015a), or bermudagrass (*Cynodon dactylon*) (Shi et al., 2013) under water deficit condition. In addition, Spm-induced accumulation of total available carbohydrates improved OA and metabolic homeostasis, which alleviated heat-induced leaf senescence (Liang et al., 2021). However, Spm-regulated specific sugars, such as monosaccharides, disaccharides, and polysaccharides, have not been well reported in plants under drought or heat stress. In the current study, the Spm jointly regulated the accumulation of mannose, maltose, and galactose in leaves of creeping bentgrass under water and heat stress. Mannose, maltose, and galactose are not only important osmolytes and energy matters but also have other potential functions for stress adaptation in plants. It has been found that mannose was a negative regulator of senescence and also responsible for the improvement in redox homeostasis and growth under water deficit or high-temperature conditions (Wang et al., 2011; Zhao et al., 2020; Guo et al., 2022). The study of Ma et al. (2014) found that drought tolerance of transgenic *Arabidopsis* overexpressing the alfalfa (*Medicago Sativa*) *GME* gene that catalyzed the conversion of GDP-D-mannose to GDP-L-galactose was significantly improved and was associated with a significant increase in antioxidant capacity. Maltose is a compatible-solute stabilizing factor that has the ability to protect proteins, membranes, and photosynthetic electron transport chains under high-temperature stress (Kaplan and Guy, 2004). Maltose also exhibited a positive effect on alleviating drought-induced wheat growth inhibition (Ibrahim and Abdellatif, 2016). These studies together with our current findings indicated that Spm-regulated tolerance to water stress or heat stress could be related to enhanced OA, osmoprotection, and antioxidant capacity by improving the accumulation of mannose, maltose, and galactose in creeping bentgrass.

Interestingly, exogenous application of Spm significantly induced a 27, 100, 19, 77, 47, or 53% increase in glucose-1-phosphate, sucrose-6-phosphate, fructose, kestose, maltotriose, or xylose content when creeping bentgrass suffered from water stress. Sucrose-6-phosphate and glucose-1-phosphate play important roles in sugar metabolism, carbon flux, and the energy cycle in plants (Engels et al., 2008; Poonam et al., 2016). Nano-zinc oxide promoted the synthesis of glucose-1-phosphate that was a benefit for drought tolerance in maize (*Zea mays*) (Sun et al., 2021). It has been found that the accumulation of fructose as a common osmolyte for OA or precursor in cellular respiration helped drought-tolerant *Thymus serpyllum* and sensitive *Thymus vulgaris* plants to tolerate drought stress (Ashrafi et al., 2018). Exogenous application of chitosan and mannose enhanced drought tolerance of white clover and creeping bentgrass associated with the accumulation of fructose and xylose (Li et al., 2017b, 2019). In addition, the study by Liu et al. (2019) found that kestose accumulation likely functioned as a storage carbohydrate that provided energy for rhizosheath formation of switchgrass (*Panicum virgatum*) under drought stress. Maltotriose was a common trisaccharide among rice genotypes in response to drought stress (Da Costa et al., 2022). Spm-induced accumulation of these sugars could play positive roles in drought tolerance, but an individual function of these sugars deserved to be further investigated in our further study.

Amino acids are critical primary metabolites for plant growth and development, reproduction, and stress adaptation (Hildebrandt Tatjana et al., 2015). A previous study proved that upregulation of Put metabolism affected the accumulation of almost all amino acids in cells (Mohapatra et al., 2010). For the possible function of various amino acids, alanine and glycine could induce *heat shock protein 70* (*HSP70*) expression in cells associated with the improvement in thermotolerance (Nissim et al., 1992). Significant increases in alanine and glycine were also related to improved heat tolerance of creeping bentgrass (Li et al., 2016a), which was consistent with our current finding in Spm-treated creeping bentgrass. It was noteworthy that Spm expressly induced the accumulation of glutamic acid, methionine, serine, and threonine in leaves of creeping bentgrass under water stress. Glutamic acid not only acts as a signal trigger and nitrogen resource for plant growth but also is involved in chlorophyll biosynthesis for photosynthetic performance (Forde and Walch-Liu, 2009; Toyota et al., 2018). Exogenous application of glutamic acid could suppress heat-induced leaf senescence and chlorophyll degradation by improving amino acid metabolism in creeping bentgrass (Stephanie et al., 2021). In addition, it was found that drought stress decreased the methionine biosynthesis pathways in plants (Larrainzar et al., 2014). However, exogenous application of methionine mitigated oxidative damage and growth retardation of bitter gourd (*growth retardation*) under drought stress (Akram et al., 2020). Enhanced accumulation of serine and threonine induced by exogenous application of chitosan improved stress adaptation of white clover to water deficit (Li et al., 2017b). In response to water stress, Spm-regulated accumulation of amino acids as mentioned above could maintain metabolic and growth homeostasis in creeping bentgrass.

In addition to sugars and amino acids, multiple organic acids were also significantly regulated by Spm in creeping bentgrass under water stress or heat stress. Pyruvic acid acts as an intermediate compound during the metabolism of carbohydrates and proteins and is also metabolized into the TCA cycle to produce energy in plants (Tovar-Méndez et al., 2003). Aconitic acid and ketoglutaric acid are two intermediates of the TCA cycle (Sweetlove et al., 2010). It was found that the maintenance of accumulation of TCA cycle intermediates was one of the important regulatory mechanisms for plant growth and stress tolerance under drought stress (Merewitz et al., 2011; Guo et al., 2018; Li et al., 2019). In the current study, water stress extremely decreased the accumulation of pyruvic acid, aconitic acid, and ketoglutaric acid in leaves of Spm-pretreated and untreated creeping bentgrass plants, but the application of Spm could significantly alleviate the deficit of these metabolites under water stress. This could indicate Spm-regulated growth and tolerance to water deficit associated with better maintenance of the TCA cycle for energy production in creeping bentgrass. However, Spm pretreatment did not enhance the accumulation of intermediates of the TCA cycle but increased the accumulation of other organic acids such as gallic acid and malic acid under heat stress. Previous studies proved that the antioxidant potency of gallic acid is associated with the amelioration of tolerance to high temperature, salt stress, and osmotic stress in plants (Ozfidan-Konakci et al., 2015; Farhoosh and Nyström, 2018). The study of Du et al. (2011) found that heat-tolerant hybrid bermudagrass (*Cynodon transvaalensis* × *Cynodon dactylon*) accumulated more malic acid than heat-sensitive Kentucky bluegrass (*Poa pratensis*) during long-term heat stress, suggesting that the malic acid could play an important role in hybrid bermudagrass adaptation to heat stress.

Other metabolites including myo-inositol, nicotinic acid, and urea were also related to stress adaptation in plants. Beneficial effects of myo-inositol on ameliorating drought damage have been widely studied in plants. For example, foliar application of myo-inositol effectively alleviated drought damage to creeping bentgrass through detoxifying reactive oxygen species (ROS) and improving OA (Li et al., 2020b). Transgenic sweet potato (*Ipomoea batatas*) plants overexpressing a *myo-inositol-1-phosphate synthase* (*MIPS*) gene encoding myo-inositol biosynthesis exhibited better drought tolerance than wild type associated with stronger ROS-scavenging system and sensitive signal transduction pathways in response to drought stress (Zhai et al., 2016). Nicotinic acid known as vitamin B3 has also been reported to be propitious to plants' survival and growth under abiotic stress. Overexpression of a *nicotinamidase 3* (*NIC3*) gene involved in nicotinic acid biosynthesis exhibited similar effects with exogenous application of nicotinic acid on improving stress tolerance and biomass of *Arabidopsis thaliana* under drought stress (Ahmad et al., 2021). Exogenous application of nicotinic acid could also effectively alleviate salinity-induced damage and biomass loss of onion (*Allium cepa*) plants (Hussein et al., 2014). In addition, urea as the main nitrogen source has dual functions of growth promotion and stress tolerance in plants (Witte, 2011). Urea application could delay the senescence of creeping bentgrass by stabilizing proteins and cell membranes under heat stress (Jespersen and Huang, 2015). These studies together with our current findings indicated that Spm-induced a 59% increase in myo-inositol under water stress, a 321% increase in nicotinic acid under heat stress, and a 184 or a 29% increase in urea under water stress or heat stress could contribute to growth maintenance and better stress tolerance in creeping bentgrass.

## Conclusion

Exogenous application of Spm could effectively alleviate growth retardant and damage effects induced by water and heat stress by increasing endogenous PAs, Put, Spd, and Spm contents. The physiological analysis found that Spm regulated water homeostasis, photosynthetic performance, oxidative damage, and cell membrane stability when creeping bentgrass responded to water or heat stress. Metabolites profiling showed that a total of 61 metabolites were differentially or commonly regulated by Spm in leaves of creeping bentgrass under water stress and heat stress. Under water stress, Spm mainly induced the accumulation of glucose-1-phosphate, sucrose-6-phosphate, fructose, kestose, maltotriose, xylose, glutamic acid, methionine, serine, threonine pyruvic acid, aconitic acid, ketoglutaric acid, and myo-inositol. In response to heat stress, the accumulation of alanine, glycine, gallic acid, malic acid, and nicotinic acid was specifically regulated by Spm. Spm also commonly enhanced the accumulation of mannose, maltose, galactose, and urea under water and heat stress. The current study provides novel evidence of global metabolites reprogramming associated with Spm-induced tolerance to water stress and heat stress in horticultural plants.

## Data availability statement

The original contributions presented in the study are included in the article/Supplementary material, further inquiries can be directed to the corresponding author.

## Author contributions

ZL: methodology, investigation, conceptualization, writing—original draft, and funding acquisition. BC and XW: methodology and investigation. YZ and GF: writing—review and editing. YP: supervision, conceptualization, writing—review and editing, and funding acquisition. All authors contributed to the article and approved the submitted version.

## Funding

This research was supported by the China Postdoctoral Science Foundation (2021T140058) and the Sichuan Forage Innovation Team Project of the Industrial System Construction of Modern Agriculture of China (sccxtd-2020-16).

## Conflict of interest

The authors declare that the research was conducted in the absence of any commercial or financial relationships that could be construed as a potential conflict of interest.

## Publisher's note

All claims expressed in this article are solely those of the authors and do not necessarily represent those of their affiliated organizations, or those of the publisher, the editors and the reviewers. Any product that may be evaluated in this article, or claim that may be made by its manufacturer, is not guaranteed or endorsed by the publisher.
