# Supplementary material for: Spermine-mediated metabolic homeostasis improves growth and stress tolerance in creeping bentgrass (Agrostis stolonifera) under water or high-temperature stress
Source: Front Plant Sci. 2022 Aug 11;13:944358. doi: 10.3389/fpls.2022.944358 (PMC9404338; doi:10.3389/fpls.2022.944358)
Supplement: Supplementary file 1 [file Presentation_1.pdf]

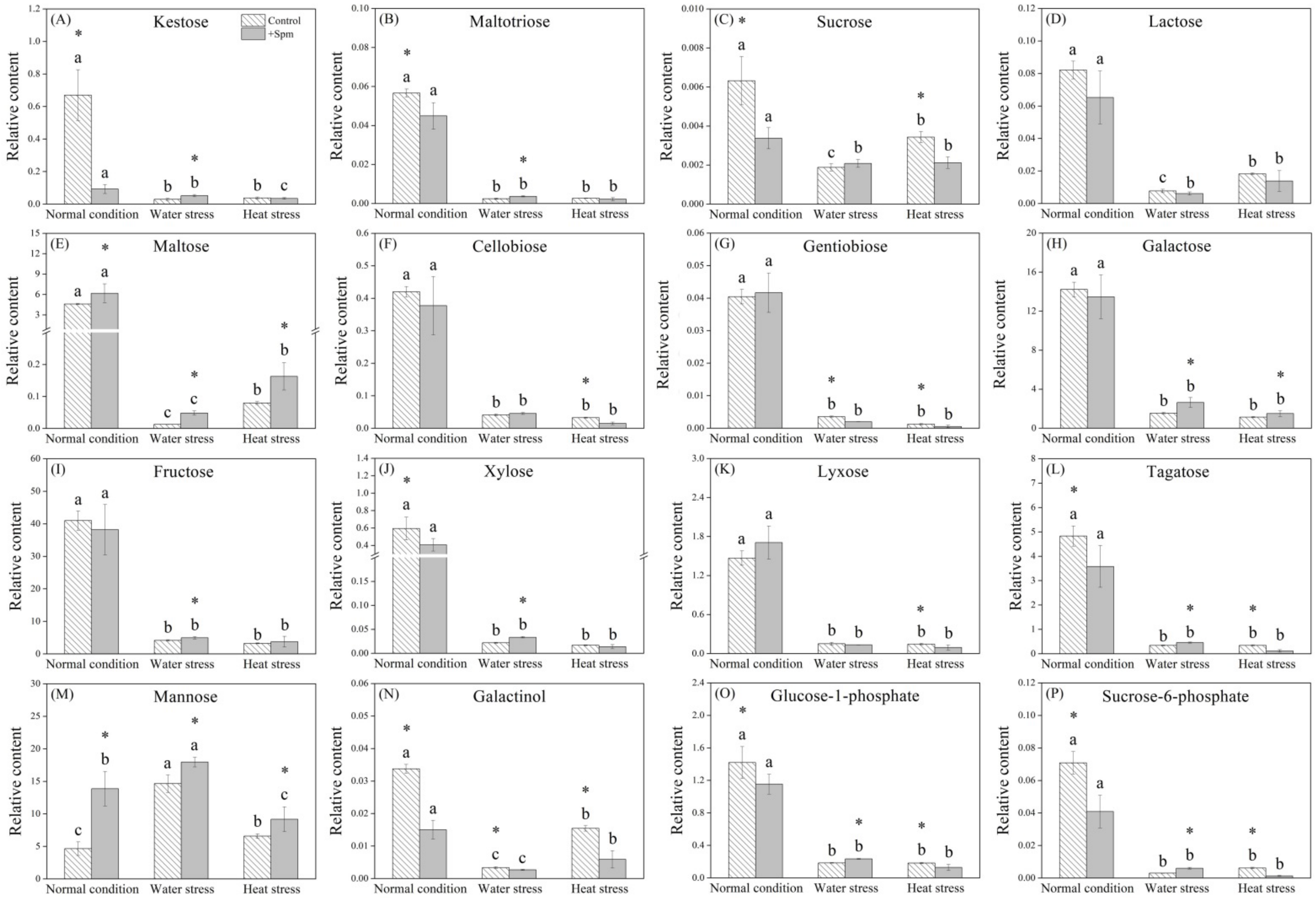

Fig. S1 Changes in relative content of 16 sugars in response to the Spm application under different conditions. Vertical bars indicate ±SE of mean (n=4). Different letters above striated or gray columns indicate significant differences for a particular treatment (Control or +Spm) for comparison among normal condition, water stress, and heat stress; the asterisk "*" indicates significant differences between the Control and the +Spm under one particular condition (normal condition, water stress, or heat stress) based on LSD (P<0.05).

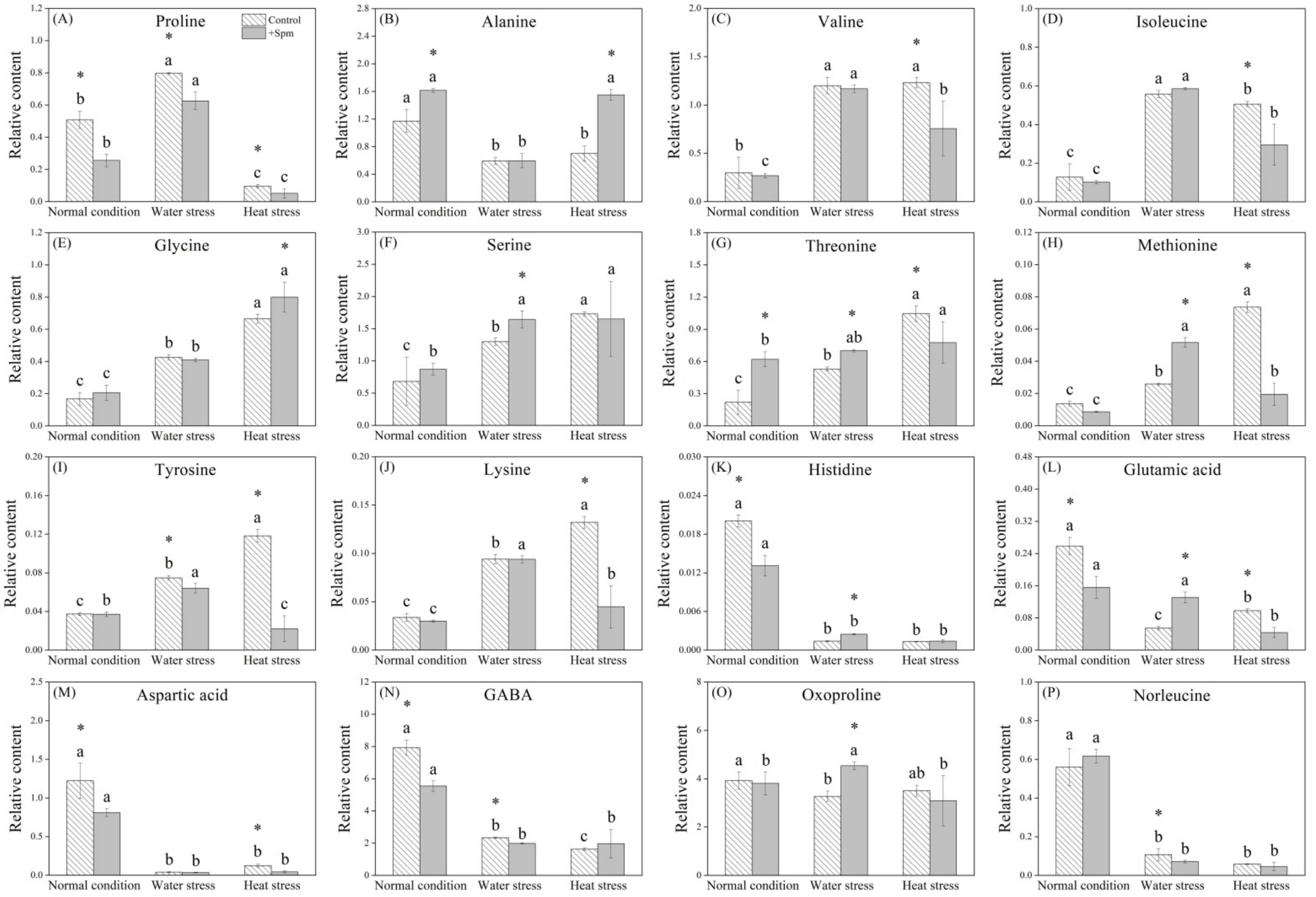

Fig. S2 Changes in relative content of 16 amino acids in response to the Spm application under different conditions. Vertical bars indicate ±SE of mean (n=4). Different letters above striated or gray columns indicate significant differences for a particular treatment (Control or +Spm) for comparison among normal condition, water stress, and heat stress; the asterisk "*" indicates significant differences between the Control and the +Spm under one particular condition (normal condition, water stress, or heat stress) based on LSD (P<0.05).

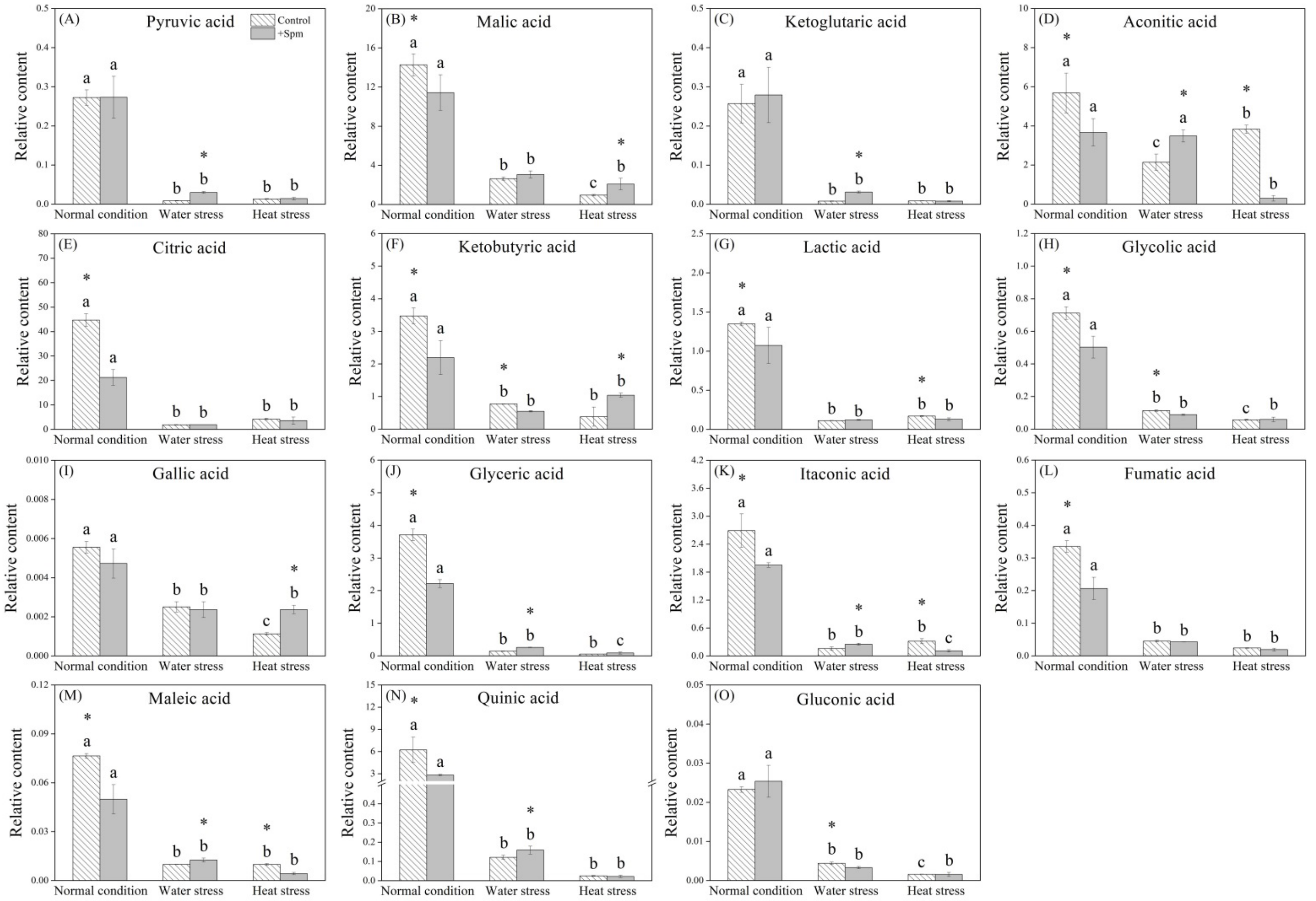

Fig. S3 Changes in relative content of 15 organic acids in response to the Spm application under different conditions. Vertical bars indicate ±SE of mean (n=4). Different letters above striated or gray columns indicate significant differences for a particular treatment (Control or +Spm) for comparison among normal condition, water stress, and heat stress; the asterisk “*” indicates significant differences between the Control and the +Spm under one particular condition (normal condition, water stress, or heat stress) based on LSD (P<0.05).

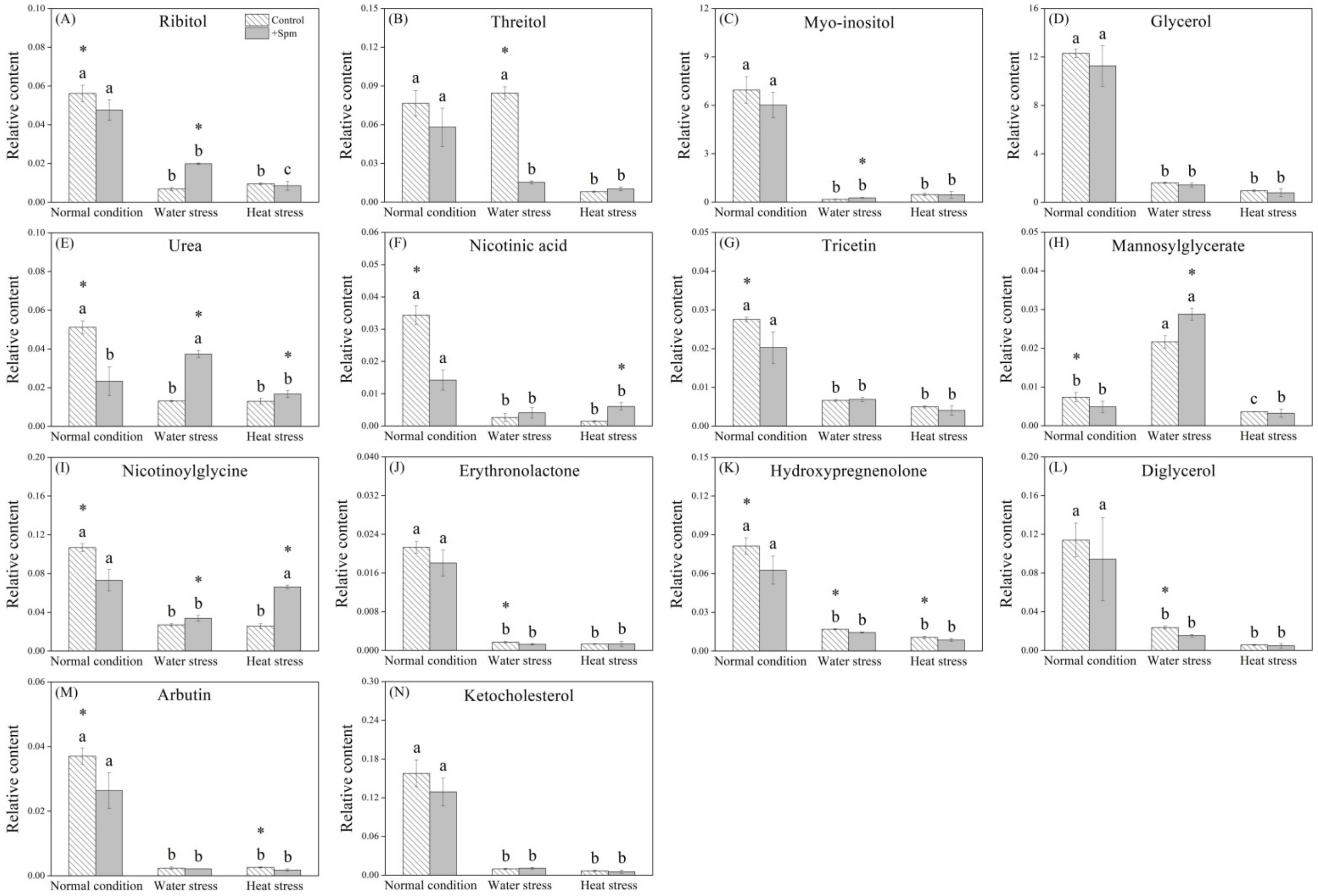

Fig. S4 Changes in relative content of 14 other metabolites in response to the Spm application under different conditions. Vertical bars indicate ±SE of mean (n=4). Different letters above striated or gray columns indicate significant differences for a particular treatment (Control or +Spm) for comparison among normal condition, water stress, and heat stress; the asterisk "*" indicates significant differences between the Control and the +Spm under one particular condition (normal condition, water stress, or heat stress) based on LSD (P<0.05).
